# PFOA/PFOS induce ferroptosis in bladder epithelial cells through inhibition of ACSL4 ubiquitination

**DOI:** 10.3389/ftox.2026.1774625

**Published:** 2026-05-11

**Authors:** Hao Xu, Zhihua Ye, Juan Liu, Mengwei Liu, Qinghua Hu, Yi Liu, Qiankun Zhang, Jian Ou, Xin Shen, Xiaogang Chen

**Affiliations:** 1 Department of Urology, Huangshi Central Hospital, Affiliated Hospital of Hubei Polytechnic University, Huangshi, Hubei, China; 2 Hubei Key Laboratory of Kidney Disease Pathogenesis and Intervention, Huangshi, Hubei, China; 3 Department of Science and Education, Huangshi Central Hospital, Affiliated Hospital of Hubei Polytechnic University, Huangshi, Hubei, China; 4 Department of Urology, Huangshi Central Hospital Graduate Joint Training Base, School of Medicine, Wuhan University of Science and Technology, Huangshi, Hubei, China; 5 Department of Medical Affairs, Huangshi Central Hospital, Affiliated Hospital of Hubei Polytechnic University, Huangshi, Hubei, China; 6 Department of Hepatobiliary, Urologic, and Gynecologic Oncology, Huangshi Central Hospital, Affiliated Hospital of Hubei Polytechnic University, Huangshi, Hubei, China

**Keywords:** ACSL4, bladder epithelial cell, ferroptosis, pfoa, PFOs, ubiquitination

## Abstract

**Introduction:**

Perfluoroalkyl and polyfluoroalkyl substances (PFAS) are persistent environmental pollutants, but their role in bladder epithelial injury remains unclear.

**Methods:**

SV-HUC-1 cells were exposed to PFOA and PFOS. Cell viability, lipid peroxidation, and ROS levels were assessed using CCK-8 assay, flow cytometry, and transmission electron microscopy. Protein expression and ubiquitination were analyzed by Western blotting and molecular docking.

**Results:**

PFOA and PFOS induced ferroptosis in bladder epithelial cells, characterized by increased lipid peroxidation and ROS levels. Mechanistically, PFOA/PFOS inhibited ACSL4 ubiquitination at K593 and K690, leading to its stabilization and promoting ferroptosis. Knockdown of ACSL4 significantly reversed these effects.

**Discussion:**

These findings reveal a novel mechanism by which PFAS induce ferroptosis via regulation of ACSL4 ubiquitination, providing new insights into PFAS-induced bladder toxicity.

## 1 Introduction

Interstitial cystitis/bladder pain syndrome (IC/BPS) is a chronic, potentially debilitating disease of unknown etiology, commonly associated with lower urinary tract symptoms characterized by pelvic pain (Hanno et al., 2015; Homma et al., 2016; van de Merwe et al., 2008). Although IC/BPS is not a life-threatening condition, it causes significant physical pain, mental distress, and financial burden for patients. Moreover, previous studies have reported a close association between IC/BPS and bladder tumors (Keller et al., 2013). The etiology of IC/BPS remains largely unclear, however, a growing body of evidence suggests that it may involve complex interactions among neural, endocrine, immune, and other mechanisms (Konkle et al., 2012; Lee et al., 2018; Kim et al., 2022). Therefore, further exploration of the underlying causes and mechanisms of IC/BPS holds significant clinical importance. Recent studies have implicated ferroptosis, characterized by iron-dependent lipid peroxidation and oxidative stress, in IC/BPS progression. For instance, oxidative stress-induced ferroptosis contributes to bladder dysfunction in IC/BPS models, with Wnt/β-catenin signaling inhibiting ferroptosis via NF-κB downregulation (Fang et al., 2024). Additionally, ferroptosis-related genes have been identified in IC/BPS datasets, suggesting a role in epithelial damage and inflammation (Jiang et al., 2022).

Perfluoroalkyl and polyfluoroalkyl substances (PFAS), including perfluorooctanoic acid (PFOA) and perfluorooctane sulfonic acid (PFOS), are a group of synthetic chemicals with widespread industrial and consumer applications due to their surfactant properties. These compounds have been used in products such as cookware, food packaging, textiles, and firefighting foams since the 1940s (Buck et al., 2011). Both PFOA and PFOS are persistent in the environment and bioaccumulate in living organisms, posing significant health risks. Despite the phase-out of PFOA and PFOS in many developed countries, they remain detectable in human biofluids and environmental media, as they are slow to degrade (Liu et al., 2020; Zhang et al., 2023). Studies have linked exposure to these chemicals to a range of health effects, including liver toxicity, reproductive dysfunction, and fetal growth retardation (Franco et al., 2020; Fei et al., 2007). Moreover, PFOS has been identified as a persistent organic pollutant and is associated with adverse effects on liver metabolism, neurotoxicity, and oxidative damage (Wang Z. et al., 2023; Wu et al., 2023). In bladder tissue, PFAS exposure has been associated with urothelial toxicity and increased cancer risk. Epidemiological studies link PFOA and PFOS to higher incidences of bladder cancer, potentially via oxidative stress and cellular damage (Yan et al., 2026). *In vitro*, PFAS disrupt urothelial cell function, including apoptosis and ferroptosis pathways (Wang et al., 2022). These findings underscore the need to investigate PFAS mechanisms in bladder epithelial cells. Despite ongoing research, the molecular mechanisms underlying PFOA and PFOS-induced toxicity, particularly in relation to cellular processes like ferroptosis, remain poorly understood. This paper explores the mechanism by which PFOA and PFOS inhibit the ubiquitination of ACSL4, leading to ferroptosis in bladder epithelial cells, providing new insights into the toxicological pathways of these persistent pollutants.

Ferroptosis is a unique form of regulated cell death characterized by iron dependence and lipid peroxidation, distinguishing it from apoptosis and other cell death pathways (Wang Y. et al., 2023). It is regulated by a balance between the generation and clearance of lipid peroxides, a crucial process for maintaining redox homeostasis in cells (Sharma et al., 2023). Ferroptosis is tightly controlled by pathways linked to amino acid, iron, and lipid metabolism, with acyl-CoA synthetase long-chain family member 4 (ACSL4) playing a central role. ACSL4 promotes the incorporation of polyunsaturated fatty acids (PUFAs) into membrane phospholipids, which are prone to peroxidation, making ACSL4 a key determinant of ferroptotic sensitivity (Liang et al., 2022). Ferroptosis has been implicated in various liver diseases, and studies have demonstrated that PFOS induces ferroptosis in hepatocytes and liver tissues (Yang et al., 2023; Ren et al., 2024). However, the detailed molecular mechanisms underlying PFOS-induced ferroptosis remain unclear.

In this study, we exposed the bladder epithelial cell line SV-HUC-1 to PFAS to investigate their toxicity to bladder epithelial cells. Both PFOA and PFOS were found to induce ferroptosis in bladder epithelial cells by inhibiting the ubiquitination of ACSL4, which led to exacerbated phospholipid peroxidation and elevated cellular ROS levels. Our findings provide new insights into mitigating the toxicity of perfluoroalkyl compounds on bladder epithelial cells.

## 2 Materials and methods

### 2.1 Cell lines

SV-HUC-1 and HEK293T cell lines were obtained from the Cell Bank of the Chinese Academy of Sciences. Cells were maintained in Dulbecco’s Modified Eagle Medium (DMEM) containing 10%–15% fetal bovine serum (FBS) under standard culture conditions. Routine *mycoplasma* testing was conducted prior to experimental use to ensure cell quality. HEK293T cells were used for transfection and ubiquitination assays, while SV-HUC-1 cells were used for all other experiments.

### 2.2 Cell viability analysis

Cell viability was assessed using a CCK-8 assay kit (Dojindo, Japan) following the manufacturer’s protocol. Cells were seeded at a density of 5 × 10^3^ cells per well in 96-well plates. After 24 h of exposure to PFOA or PFOS (0–100 μM), cell viability was assessed using the CCK-8 assay. Briefly, the CCK-8 reagent was added to each well, and the plates were incubated at room temperature for approximately 1 h before measurement.

### 2.3 Western blotting

Cells were rinsed three times with ice-cold PBS and lysed in NP-40 buffer containing a protease inhibitor cocktail (Beyotime) for 30 min at 4 °C. Protein concentrations were quantified using a Coomassie Brilliant Blue G (Acid Blue 90) protein assay kit (Beyotime). Equal amounts of protein were separated by SDS–PAGE and subsequently transferred onto PVDF membranes. Following blocking with 5% non-fat milk, membranes were incubated with specific primary antibodies overnight at 4 °C. Afterward, they were exposed to horseradish peroxidase–conjugated secondary antibodies (1:5,000 dilution) for 2 h at room temperature (25 °C). Protein bands were finally detected using an enhanced chemiluminescence detection kit (Thermo Fisher Scientific).

### 2.4 Antibodies and reagents

The specific antibodies and reagents used in this study were as follows: Antibody against Tubulin was used as control (Proteintech, Cat #10068-1-AP). GPX4 (Proteintech, Cat #67763-1-Ig), FSP1 (Proteintech, Cat #68049-1-Ig), ACSL4 (Proteintech, Cat #66617-1-Ig), Ub (Cell Signaling Technology, Cat#3933) were used for Western blot assays. PFOA (Cat #355-67-1) and PFOS (Cat #33607) were purchased from Sigma.

### 2.5 Quantitative RT-PCR

Total RNA was extracted using TRIzol reagent (TaKaRa, China), and complementary DNA (cDNA) was synthesized with a reverse transcription kit (TaKaRa) according to the manufacturer’s protocol. Subsequently, RT-PCR was conducted to assess gene expression levels. The expression of β-actin served as an internal control for normalization of target gene expression.

### 2.6 Molecular docking

The core target protein structure was retrieved from the RCSB Protein Data Bank (https://www.rcsb.org/). To further examine the molecular interactions between PFOA/PFOS and the core protein, molecular docking was conducted to predict their potential binding conformations and affinities. In particular, docking analyses were performed using the CB-Dock2 platform (Liu et al., 2022; Yang et al., 2022) (https://cadd.labshare.cn/cb-dock2/php/index.php), which allowed assessment of the binding potential of PFOA/PFOS with the identified targets, thereby providing additional support for the findings derived from the network analysis.

### 2.7 Lipid peroxidation and ROS assay

Lipid peroxidation and ROS were assessed by flow cytometry using the fluorescent probe BODIPY-C11. Cells were treated with 50 μM PFOA or PFOS for 24 h prior to BODIPY-C11 staining. Briefly, cells were plated in six-well plates at a density of 2.5 × 10^5^ cells per well and allowed to adhere overnight. The following day, cells were treated with either DMSO or Sorafenib for 24 h, after which they were incubated in 2 mL of culture medium containing 5 µM BODIPY-C11 (Thermo Fisher) for 20 min at 37 °C. Subsequently, the cells were washed with PBS to eliminate residual dye, resuspended in 500 µL PBS, and passed through a 0.4 µm nylon mesh to obtain a single-cell suspension. Flow cytometric analysis was then performed, with a minimum of 10,000 events collected for each condition.

### 2.8 Statistical analysis

All assays were performed with at least three biological replicates (independent experiments) and three technical replicates per condition, unless otherwise specified. All quantitative data are expressed as mean ± SD or mean ± SEM, as appropriate. Statistical analyses were conducted using GraphPad Prism 9.0 and SPSS version 20.0. Comparisons between two groups were performed using two-tailed Student’s t-tests, whereas multiple-group comparisons were assessed by one-way ANOVA. A P value <0.05 was considered to denote statistical significance.

## 3 Results

### 3.1 PFOA/PFOS promote human bladder epithelial cells injury

**FIGURE 1:**
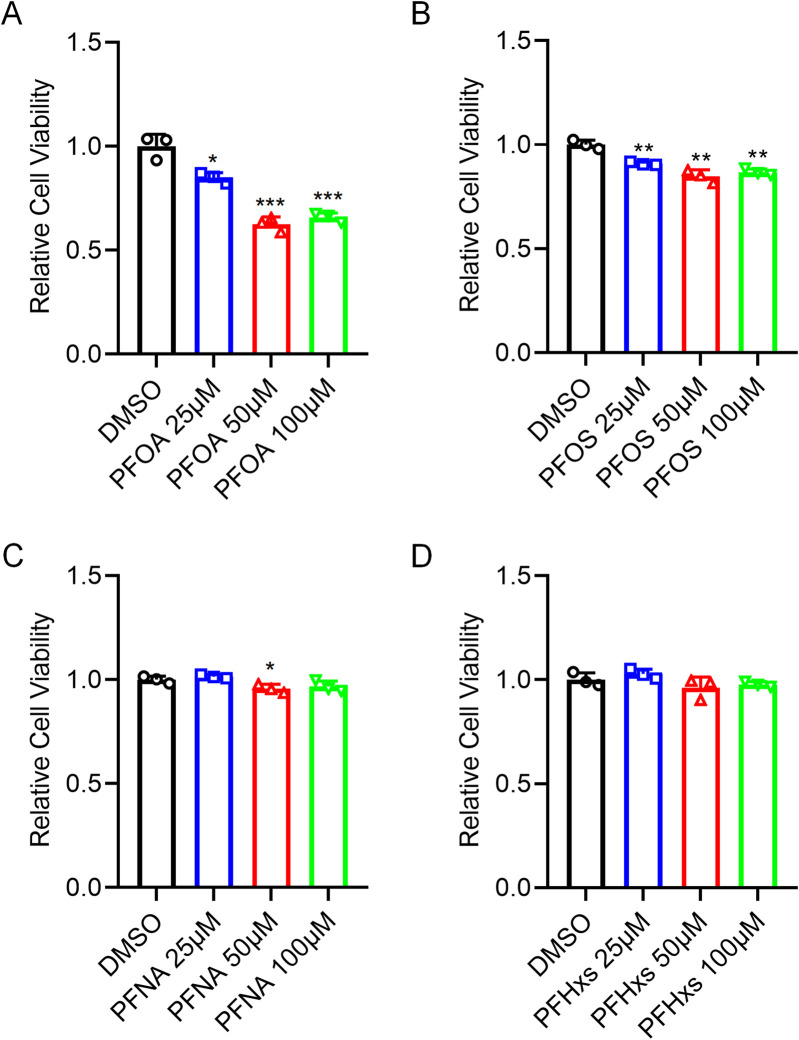
PFOA/PFOS promote human bladder epithelial cells injury. **(A–D)** Cell viability of SV-HUC-1 cells after exposure to different concentrations (0, 25, 50, and 100 μM) of PFOA **(A)**, PFOS **(B)**, PFNA **(C)**, or PFHxS **(D)** for 24 h, as determined by CCK-8 assay. *p < 0.05; **p < 0.01; ***p < 0.001; DMSO, dimethyslsulfoxide; PFOA, perfluorooctanoic acid; PFOS, perfluorooctane sulfonic acid; PFNA, Perfluorononanoic acid; PFHxs, Perfluorohexane sulfonic acid.

To investigate the effects of PFOA and PFOS on the viability of human bladder epithelial cells, SV-HUC-1 cells were exposed to PFOA or PFOS for 24 h, followed by assessment of cell viability. CCK-8 assays revealed that both PFOA and PFOS significantly suppressed the viability of SV-HUC-1 cells compared to the control group. Moreover, the inhibitory effects of PFOA and PFOS on cell viability were found to be dose-dependent (Figures 1A,B). In contrast, exposure to other PFAS compounds, including PFNA and PFHxS, did not affect the viability of SV-HUC-1 cells (Figures 1C,D). These results suggest that PFOA and PFOS specifically impair cell viability and promote cell death in human bladder epithelial cells.

### 3.2 PFOA/PFOS induce ferroptosis in human bladder epithelial cells

**FIGURE 2:**
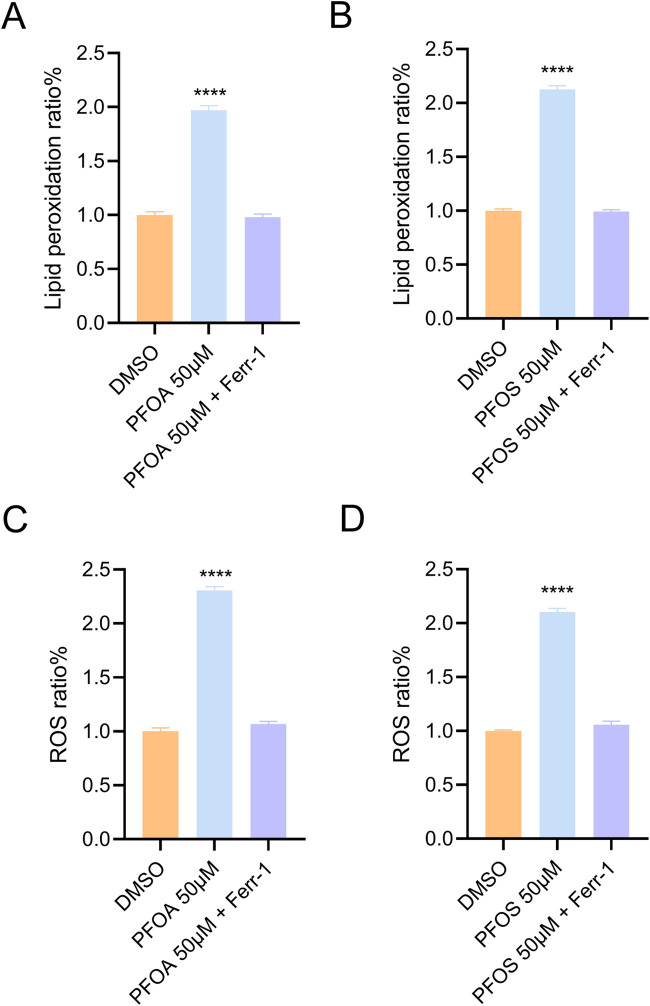
PFOA/PFOS induce ferroptosis in human bladder epithelial cells. **(A,B)** Lipid peroxidation levels in SV-HUC-1 cells treated with 50 μM PFOA or PFOS for 24 h, as determined by flow cytometry. **(C,D)** Intracellular ROS levels in SV-HUC-1 cells after treatment with 50 μM PFOA or PFOS for 24 h, detected by flow cytometry. ****p < 0.0001; Ferr-1, Ferrstaining-1.

As lipid peroxidation of cell membrane phospholipids is a hallmark of ferroptosis, we examined whether PFOA/PFOS induce ferroptosis in bladder epithelial cells by assessing lipid peroxidation levels using flow cytometry. BODIPY 581/591 staining revealed a significant increase in lipid peroxidation in SV-HUC-1 cells after 24 h of exposure to PFOA or PFOS, which was effectively reversed by the ferroptosis inhibitor ferrostatin-1 (Ferr-1) (Figures 2A,B). In addition, reactive oxygen species (ROS) levels were elevated in SV-HUC-1 cells following PFOA/PFOS exposure, showing similar trends (Figures 2C,D). These findings indicate that PFOA and PFOS promote lipid peroxidation and induce ferroptosis in human bladder epithelial cells.

### 3.3 PFOA/PFOS promote ferroptosis by upregulating ACSL4

**FIGURE 3:**
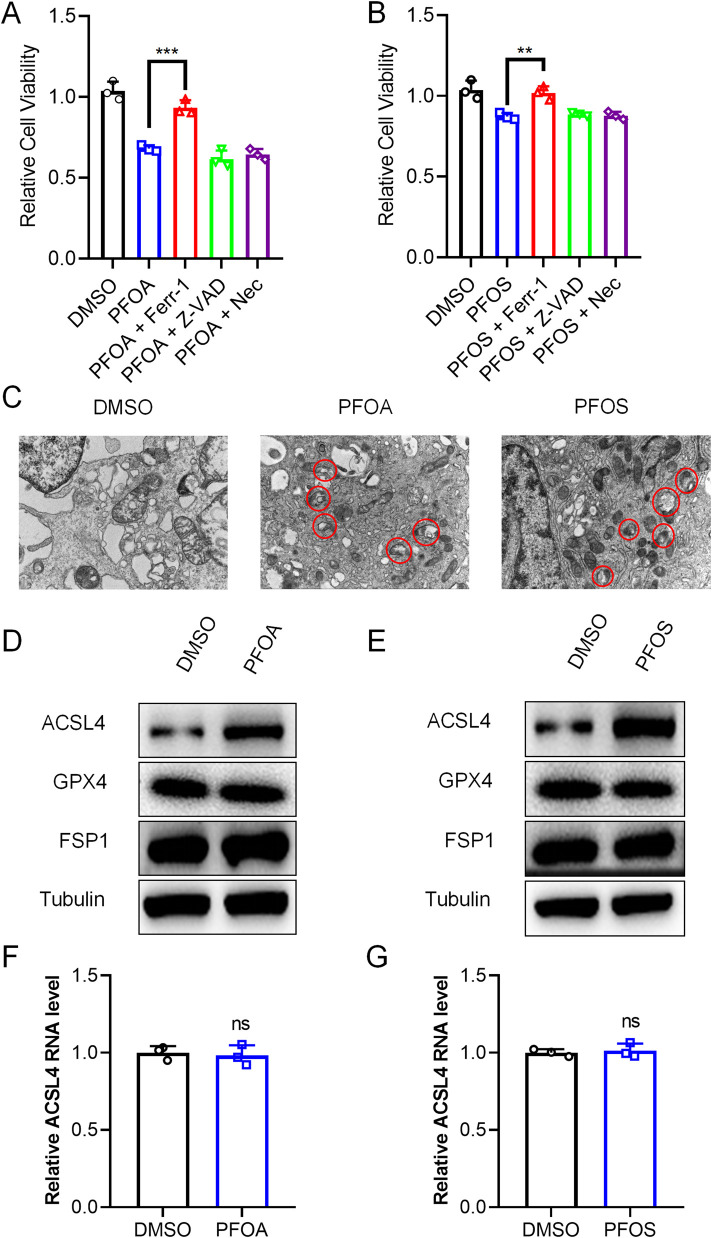
PFOA/PFOS promote ferroptosis by upregulating ACSL4. **(A,B)** Cell viability of SV-HUC-1 cells treated with 50 μM PFOA or PFOS in the presence or absence of Ferr-1, Z-VAD, or Nec for 24 h, as determined by CCK-8 assay. **(C)** Transmission electron microscopy images showing mitochondrial morphological changes in SV-HUC-1 cells after treatment with 50 μM PFOA or PFOS for 24 h **(D,E)** Western blot analysis of ACSL4, GPX4, and FSP1 protein expression in SV-HUC-1 cells treated with 50 μM PFOA or PFOS for 24 h **(F,G)** ACSL4 mRNA levels were analyzed by qRT-PCR experiments in SV-HUC-1 cells treated with 50 μM PFOA or PFOS for 24 h ns, no significance; **p < 0.01; ***p < 0.001; Nec, Necrostatin-1.

To determine whether PFOA/PFOS play a critical role in ferroptosis in bladder epithelial cells, we treated SV-HUC-1 cells with PFOA or PFOS followed by the addition of inhibitors targeting different forms of cell death. The results showed that PFOA/PFOS significantly increased cell death, which was effectively rescued by the ferroptosis inhibitor ferrostatin-1 (Ferr-1), but not by the apoptosis inhibitor Z-VAD or the necroptosis inhibitor Necrostatin-1 (Nec) (Figures 3A,B).

Given that disruption of the mitochondrial outer membrane is a key feature of ferroptosis, we used transmission electron microscopy (TEM) to examine mitochondrial morphology. TEM analysis revealed pronounced rupture of the mitochondrial outer membrane in SV-HUC-1 cells treated with PFOA or PFOS (Figure 3C).

To further explore the molecular mechanisms by which PFOA/PFOS induce ferroptosis in bladder epithelial cells, we assessed the expression of ferroptosis-related markers using Western blot analysis. The results showed that PFOA/PFOS markedly increased the protein levels of ACSL4, a key regulator of ferroptosis, without significantly affecting the expression of GPX4 or FSP1 (Figures 3D,E). However, the transcriptional level of ACSL4 remained unchanged. (Figures 3F,G). Collectively, these findings suggest that PFOA and PFOS promote ferroptosis in bladder epithelial cells by upregulating ACSL4 expression.

### 3.4 Molecular docking of PFOA/PFOS with ACSL4

**FIGURE 4:**
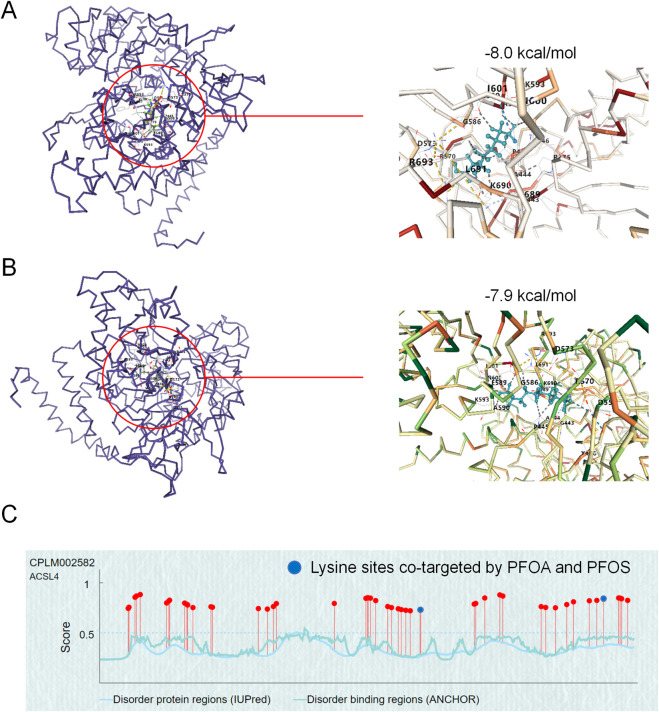
Molecular docking of PFOA/PFOS with ACSL4. **(A,B)** The protein structure of ACSL4 is represented by a blue line, with the molecular structures of PFOA and PFOS depicted as colored sticks. In the right panel, PFOA and PFOS are shown as light blue sticks, with the interacting amino acid residues labeled in black text. **(C)** Predicted ubiquitination sites of ACSL4, with blue dots indicating the lysine residues where both PFOA and PFOS bind, as shown in panels A and B.

To assess the binding potential of PFOA/PFOS with the key target protein ACSL4(PDBID:6NJS), molecular docking simulations were performed. According to the CB-Dock2 docking results, PFOA and PFOS exhibited binding energies of −8.0 kcal/mol and −7.9 kcal/mol, respectively (Figures 4A,B), with the target protein ACSL4. A binding energy value below 0 suggests potential molecular interaction, with values under −5.0 kcal/mol indicating high-affinity binding. More negative values correlate with stronger binding forces. Furthermore, CB-Dock2 docking predicted two common ubiquitination sites on ACSL4, K593 and K690, modified by PFOA/PFOS (Figure 4C). The findings revealed a high binding affinity between PFOA/PFOS and ACSL4, implying that the interaction occurs spontaneously and highlighting the pivotal role of ACSL4 in the molecular pathways underlying PFOA/PFOS-induced ferroptosis.

### 3.5 PFOA/PFOS enhance the protein stability of ACSL4 by promoting deubiquitination at the K593 and K690 sites

**FIGURE 5:**
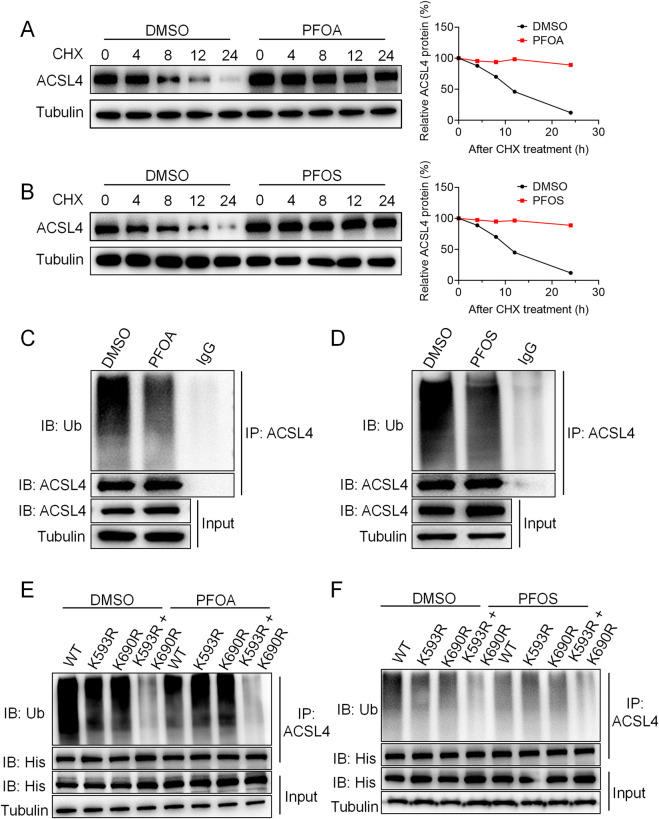
PFOA/PFOS enhance the protein stability of ACSL4 by promoting deubiquitination at the K593 and K690 sites. **(A,B)** Western blot analysis of ACSL4 protein levels in SV-HUC-1 cells treated with 50 μM CHX or 50 μM PFOA or PFOS for 0, 4, 8, 12, and 24 h. Right panels show quantification of the Western blot results. **(C,D)** Ubiquitination levels of endogenous ACSL4 in SV-HUC-1 cells after treatment with 50 μM PFOA or PFOS for 24 h, as determined by Western blot. **(E,F)** 293T cells were transfected with His-tagged ACSL4 wild-type (WT), K593R, K690R, or double mutant (K593R + K690R) constructs, treated with 50 μM PFOA or PFOS, and His-ACSL4 ubiquitination levels were analyzed by Western blot.

In our previous studies, we demonstrated that PFOA and PFOS promote ACSL4 protein expression. We hypothesized that PFOA/PFOS may regulate ACSL4 stability through the ubiquitin–proteasome pathway, and this was tested by treating SV-HUC-1 cells with 50 μg/mL cycloheximide (CHX), a protein synthesis inhibitor. Following CHX treatment, the half-life of ACSL4 was significantly extended in cells exposed to PFOA or PFOS (Figures 5A,B).

Furthermore, we confirmed that PFOA/PFOS suppress the ubiquitination levels of ACSL4 (Figures 5C,D). To further elucidate the molecular mechanism by which PFOA/PFOS regulate ACSL4 ubiquitination, we constructed two lysine-to-arginine (K-to-R) mutant plasmids. His-tagged wild-type ACSL4 (His-ACSL4-WT), K593R and K690R mutants were stably expressed in SV-HUC-1 cells under CHX treatment and PFOA/PFOS exposure, and the ubiquitination levels of ACSL4 were assessed. Co-immunoprecipitation (Co-IP) assays revealed that both K593R and K690R mutations reduced the ubiquitination of ACSL4. Interestingly, simultaneous expression of both K593R and K690R mutants resulted in a more pronounced suppression of ACSL4 ubiquitination (Figures 5E,F). These results indicate that PFOA and PFOS enhance ACSL4 stability by promoting deubiquitination at the K593 and K690 sites.

### 3.6 PFOA/PFOS induces ferroptosis in human bladder epithelial cells by promoting the protein stability of ACSL4

**FIGURE 6:**
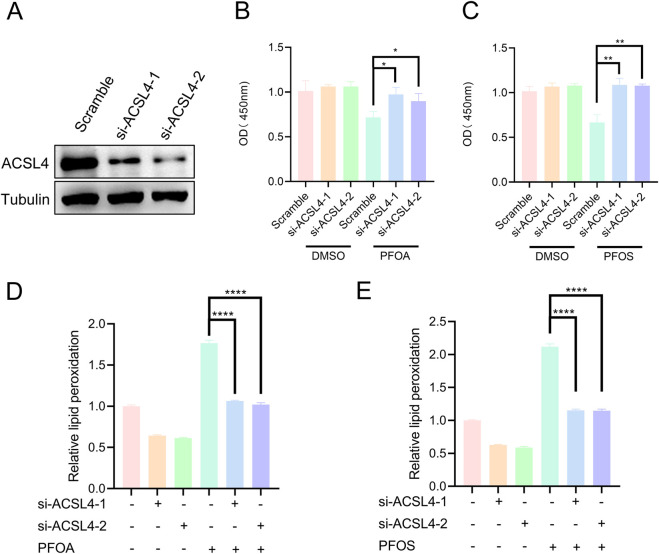
PFOA/PFOS induces ferroptosis in human bladder epithelial cells by promoting the protein stability of ACSL4. **(A)** Western blot analysis of ACSL4 protein expression in SV-HUC-1 cells transfected with ACSL4 siRNA. **(B,C)** Cell viability of the above cells treated with 50 μM PFOA or PFOS for 24 h, as determined by CCK-8 assay. **(D,E)** Lipid peroxidation levels in SV-HUC-1 cells transfected with ACSL4 siRNA and treated with 50 μM PFOA or PFOS for 24 h, measured by flow cytometry.

**FIGURE 7:**
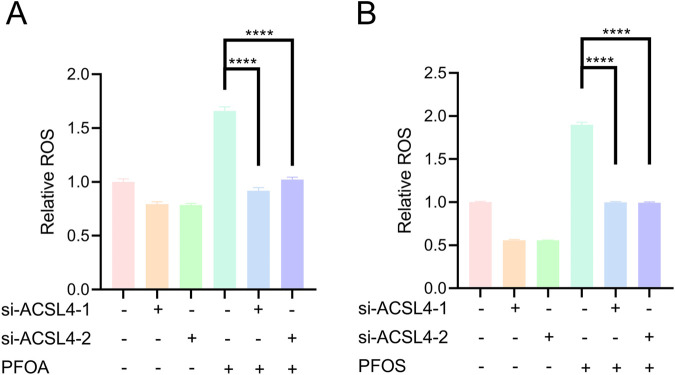
PFOA/PFOS induces ferroptosis in human bladder epithelial cells by promoting the protein stability of ACSL4. **(A,B)** Intracellular ROS levels in SV-HUC-1 cells transfected with ACSL4 siRNA and treated with 50 μM PFOA or PFOS for 24 h, as measured by flow cytometry.

In our previous experiments, we demonstrated that PFOA and PFOS promote ACSL4 protein stability by enhancing its deubiquitination at the K593 and K690 sites. To further investigate the role of the interaction between PFOA/PFOS and ACSL4 in ferroptosis, we knocked down ACSL4 expression in bladder epithelial cells following PFOA/PFOS exposure (Figure 6A). The results showed that PFOA/PFOS exposure significantly reduced cell viability, whereas silencing ACSL4 effectively restored cell viability (Figures 6B,C). We then performed lipid peroxidation assays and found that lipid peroxidation levels were elevated in bladder epithelial cells exposed to PFOA/PFOS, while ACSL4 knockdown significantly suppressed this increase (Figures 6D,E). Consistent results were obtained in the ROS assays (Figures 7A,B). Together, these findings indicate that PFOA and PFOS mediate ferroptosis in bladder epithelial cells by stabilizing ACSL4.

## 4 Discussion

PFOA and PFOS are two major PFAS widely present in the environment due to their chemical stability and resistance to degradation. Mounting epidemiological and toxicological evidence suggests a close association between PFAS exposure and urogenital diseases, including bladder disorders and carcinogenesis (Steenland et al., 2010; Su et al., 2019; Park, 2024). However, the precise molecular mechanisms underlying PFAS-induced bladder epithelial injury remain incompletely understood. In this study, we reveal that PFOA and PFOS induce ferroptosis in human bladder epithelial cells by promoting the protein stability of ACSL4 via deubiquitination at lysine residues K593 and K690.

Our initial findings demonstrated that PFOA and PFOS significantly suppressed the viability of SV-HUC-1 cells in a dose-dependent manner, whereas other PFAS analogs such as PFNA and PFHxS had negligible effects. This highlights the unique cytotoxic profile of PFOA and PFOS in bladder epithelial cells. The lack of toxicity from PFNA and PFHxS may stem from structure-activity relationships: shorter-chain PFAS exhibit lower cytotoxicity due to reduced bioaccumulation and cellular uptake compared to longer-chain PFOA/PFOS (Gorrochategui et al., 2014). Sulfonated PFAS (PFOS) may also be more toxic than carboxylated ones of similar length (Louisse et al., 2020). Similar trends occur in other models, like hepatocytes, where chain length correlates with potency. Further analysis revealed that PFOA/PFOS exposure elevated lipid peroxidation and ROS levels—two hallmark features of ferroptosis (Dixon and Stockwell, 2019). Importantly, these effects were reversed by ferrostatin-1, but not by inhibitors of apoptosis or necroptosis, confirming the specificity of ferroptotic cell death in this context.

A key molecular event in ferroptosis is the accumulation of lipid hydroperoxides, catalyzed by enzymes such as ACSL4, which preferentially ligates polyunsaturated fatty acids into phospholipids, rendering them susceptible to peroxidation (Doll et al., 2017; Kagan et al., 2017; Yuan et al., 2016). Our study demonstrated that PFOA/PFOS significantly increased ACSL4 protein levels, without affecting GPX4 or FSP1, two canonical anti-ferroptotic regulators. This suggests a selective upregulation of the pro-ferroptotic machinery. Through molecular docking, we identified that both PFOA and PFOS bind ACSL4 with high affinity and target conserved ubiquitination sites (K593, K690). Biochemically, we confirmed that PFOA/PFOS inhibited ACSL4 ubiquitination and enhanced its stability, a mechanism further validated using K-to-R site-directed mutants and CHX-chase experiments.

Functionally, silencing ACSL4 mitigated PFOA/PFOS-induced cytotoxicity and lipid peroxidation, reinforcing the pivotal role of ACSL4 in mediating PFAS-induced ferroptosis. These results provide the first mechanistic evidence that environmental PFAS pollutants may exert cytotoxic effects via modulating protein ubiquitination pathways, thereby linking environmental toxicology with ferroptosis biology.

Previous studies have implicated ferroptosis in various renal and hepatic toxicities associated with PFAS exposure (Ren et al., 2024; Yang et al., 2025; Yan et al., 2025). Prior studies implicated ferroptosis in PFAS toxicities: PFOS induces ferroptosis in hepatocytes via autophagy-MCU and in kidney cells via ER stress (Ren et al., 2024; Yan et al., 2025). PFOA triggers ferroptosis in hepatocytes through oxidative stress/AKT pathways (Feng et al., 2025). Our findings extend this to bladder cells, identifying ACSL4 deubiquitination as a novel mechanism, differing from liver/kidney models by focusing on epithelial-specific ubiquitination sites. However, their relevance to bladder pathology has been rarely addressed. Notably, recent clinical studies have reported increased incidence of bladder cancer in populations with long-term PFAS exposure (Keller et al., 2013; Vieira et al., 2013). While our model focuses on non-tumor bladder epithelial cells, the ferroptosis-inducing property of PFOA/PFOS raises questions about their potential to trigger chronic epithelial damage and inflammation, which may contribute to malignant transformation over time. Our findings extend prior observations by establishing a causal, mechanistic link between PFAS exposure, ACSL4 stabilization, and ferroptosis in human bladder epithelial cells.

Despite these insights, our study has several limitations. First, while SV-HUC-1 cells serve as a widely used *in vitro* model, they may not fully recapitulate the physiological complexity of human bladder tissue. Second, although we identified key ubiquitination sites regulating ACSL4 stability, the specific E3 ligase or deubiquitinase (DUB) responsible for this process remains to be elucidated. Third, *in vivo* validation in animal models of PFAS exposure is necessary to confirm the relevance of our findings in a physiological context.

Our *in vitro* concentrations (up to 100 μM) exceed environmental levels (ng/L-μg/L in water), but are standard for mechanistic studies to elicit detectable effects (Antonopoulou et al., 2024). Future *in vivo* validation at lower doses is needed.

## 5 Conclusion

In summary, our study demonstrates that PFOA and PFOS induce ferroptosis in human bladder epithelial cells through inhibition of ACSL4 ubiquitination at K593 and K690 sites, leading to protein stabilization and enhanced lipid peroxidation. This provides a novel mechanistic insight into PFAS-induced bladder epithelial toxicity. Given emerging evidence linking ferroptosis and oxidative stress to the pathogenesis of interstitial cystitis/bladder pain syndrome (IC/BPS), our findings suggest that PFAS exposure may contribute to bladder pathologies such as IC/BPS by promoting ferroptotic damage and chronic inflammation, potentially exacerbating lower urinary tract symptoms and increasing risks associated with bladder tumorigenesis. Future research should validate these mechanisms *in vivo*, explore therapeutic interventions targeting ACSL4 stability or ferroptosis inhibitors, and investigate the broader implications for bladder health in populations with environmental PFAS exposure. These efforts could inform strategies for early detection, prevention, and personalized management of PFAS-related urological disorders.

## Data Availability

The raw data supporting the conclusions of this article will be made available by the authors, without undue reservation.

## References

[B1] Antonopoulou M. Spyrou A. Tzamaria A. Efthimiou I. Triantafyllidis V. (2024). Current state of knowledge of environmental occurrence, toxic effects, and advanced treatment of PFOS and PFOA. Sci. Total Environ. 913, 169332. 10.1016/j.scitotenv.2023.169332 38123090

[B2] Buck R. C. Franklin J. Berger U. Conder J. M. Cousins I. T. de Voogt P. (2011). Perfluoroalkyl and polyfluoroalkyl substances in the environment: terminology, classification, and origins. Integr. Environ. Assess. Manag. 7 (4), 513–541. 10.1002/ieam.258 21793199 PMC3214619

[B3] Dixon S. J. Stockwell B. R. (2019). The hallmarks of ferroptosis. Annu. Rev. Cancer Biol. 3, 35–54. 10.1146/annurev-cancerbio-030518-055844 41613499 PMC12851585

[B4] Doll S. Proneth B. Tyurina Y. Y. Panzilius E. Kobayashi S. Ingold I. (2017). ACSL4 dictates ferroptosis sensitivity by shaping cellular lipid composition. Nat. Chem. Biol. 13 (1), 91–98. 10.1038/nchembio.2239 27842070 PMC5610546

[B5] Fang W. Song X. Li H. Meng F. Lv T. Huang J. (2024). Wnt/beta-catenin signaling inhibits oxidative stress-induced ferroptosis to improve interstitial cystitis/bladder pain syndrome by reducing NF-kappaB. Biochim. Biophys. Acta Mol. Cell Res. 1871 (7), 119766. 10.1016/j.bbamcr.2024.119766 38823528

[B6] Fei C. McLaughlin J. K. Tarone R. E. Olsen J. (2007). Perfluorinated chemicals and fetal growth: a study within the Danish National Birth Cohort. Environ. Health Perspect. 115 (11), 1677–1682. 10.1289/ehp.10506 18008003 PMC2072850

[B7] Feng Y. Lu B. Huang Y. Wang H. Xu J. Lin N. (2025). Perfluorooctanoic acid induces ferroptosis in hepatocytes via oxidative stress and AKT/GSK3beta/beta-Catenin pathway disruption. ACS Omega 10 (3), 2575–2585. 10.1021/acsomega.4c07198 39895706 PMC11780420

[B8] Franco M. E. Sutherland G. E. Fernandez-Luna M. T. Lavado R. (2020). Altered expression and activity of phase I and II biotransformation enzymes in human liver cells by perfluorooctanoate (PFOA) and perfluorooctane sulfonate (PFOS). Toxicology 430, 152339. 10.1016/j.tox.2019.152339 31809754

[B9] Gorrochategui E. Perez-Albaladejo E. Casas J. Lacorte S. Porte C. (2014). Perfluorinated chemicals: differential toxicity, inhibition of aromatase activity and alteration of cellular lipids in human placental cells. Toxicol. Appl. Pharmacol. 277 (2), 124–130. 10.1016/j.taap.2014.03.012 24680846

[B10] Hanno P. M. Erickson D. Moldwin R. Faraday M. M. American Urological A. (2015). Diagnosis and treatment of interstitial cystitis/bladder pain syndrome: AUA guideline amendment. J. Urol. 193 (5), 1545–1553. 10.1016/j.juro.2015.01.086 25623737

[B11] Homma Y. Ueda T. Tomoe H. Lin A. T. Kuo H. C. Lee M. H. (2016). Clinical guidelines for interstitial cystitis and hypersensitive bladder updated in 2015. Int. J. Urol. 23 (7), 542–549. 10.1111/iju.13118 27218442

[B12] Jiang Y. Zhu X. Al-Danakh A. Y. Chen Q. Yang D. (2022). Identification of immune-related genes and small-molecule drugs in interstitial Cystitis/Bladder pain syndrome based on the integrative machine learning algorithms and molecular docking. J. Immunol. Res. 2022, 2069756. 10.1155/2022/2069756 36619718 PMC9812613

[B13] Kagan V. E. Mao G. Qu F. Angeli J. P. Doll S. Croix C. S. (2017). Oxidized arachidonic and adrenic PEs navigate cells to ferroptosis. Nat. Chem. Biol. 13 (1), 81–90. 10.1038/nchembio.2238 27842066 PMC5506843

[B14] Keller J. Chiou H. Y. Lin H. C. (2013). Increased risk of bladder cancer following diagnosis with bladder pain syndrome/interstitial cystitis. Neurourol. Urodyn. 32 (1), 58–62. 10.1002/nau.22283 22826002

[B15] Kim M. M. Harvey J. Gusev A. Norton J. M. Miran S. Bavendam T. (2022). A scoping review of the economic burden of non-cancerous genitourinary conditions. Urology 166, 29–38. 10.1016/j.urology.2021.10.008 34688770

[B16] Konkle K. S. Berry S. H. Elliott M. N. Hilton L. Suttorp M. J. Clauw D. J. (2012). Comparison of an interstitial cystitis/bladder pain syndrome clinical cohort with symptomatic community women from the RAND Interstitial Cystitis Epidemiology study. J. Urol. 187 (2), 508–512. 10.1016/j.juro.2011.10.040 22177158 PMC3894739

[B17] Lee M. H. Chang K. M. Tsai W. C. (2018). Morbidity rate and medical utilization in interstitial cystitis/painful bladder syndrome. Int. Urogynecol J. 29 (7), 1045–1050. 10.1007/s00192-018-3574-x 29532129

[B18] Liang L. Pan Y. Bin L. Liu Y. Huang W. Li R. (2022). Immunotoxicity mechanisms of perfluorinated compounds PFOA and PFOS. Chemosphere 291 (Pt 2), 132892. 10.1016/j.chemosphere.2021.132892 34780734

[B19] Liu Y. Li A. Buchanan S. Liu W. (2020). Exposure characteristics for congeners, isomers, and enantiomers of perfluoroalkyl substances in mothers and infants. Environ. Int. 144, 106012. 10.1016/j.envint.2020.106012 32771830

[B20] Louisse J. Rijkers D. Stoopen G. Janssen A. Staats M. Hoogenboom R. (2020). Perfluorooctanoic acid (PFOA), perfluorooctane sulfonic acid (PFOS), and perfluorononanoic acid (PFNA) increase triglyceride levels and decrease cholesterogenic gene expression in human HepaRG liver cells. Arch. Toxicol. 94 (9), 3137–3155. 10.1007/s00204-020-02808-0 32588087 PMC7415755

[B21] Park R. M. (2024). Risk assessment for perfluorooctanoic acid (PFOA) in air, blood serum and water: mortality from liver and kidney disease. Occup. Environ. Med. 81 (7), 373–380. 10.1136/oemed-2023-109228 39025495

[B22] Ren S. Wang J. Dong Z. Li J. Ma Y. Yang Y. (2024). Perfluorooctane sulfonate induces ferroptosis-dependent non-alcoholic steatohepatitis via autophagy-MCU-caused mitochondrial calcium overload and MCU-ACSL4 interaction. Ecotoxicol. Environ. Saf. 280, 116553. 10.1016/j.ecoenv.2024.116553 38850699

[B23] Sharma P. Nandave M. Nandave D. Yadav S. Vargas-De-La-Cruz C. Singh S. (2023). Reactive oxygen species (ROS)-mediated oxidative stress in chronic liver diseases and its mitigation by medicinal plants. Am. J. Transl. Res. 15 (11), 6321–6341. 38074830 PMC10703659

[B24] Steenland K. Fletcher T. Savitz D. A. (2010). Epidemiologic evidence on the health effects of perfluorooctanoic acid (PFOA). Environ. Health Perspect. 118 (8), 1100–1108. 10.1289/ehp.0901827 20423814 PMC2920088

[B25] Sunderland E. M. Hu X. C. Dassuncao C. Tokranov A. K. Wagner C. C. Allen J. G. (2019). A review of the pathways of human exposure to poly- and perfluoroalkyl substances (PFASs) and present understanding of health effects. J. Expo. Sci. Environ. Epidemiol. 29 (2), 131–147. 10.1038/s41370-018-0094-1 30470793 PMC6380916

[B26] van de Merwe J. P. Nordling J. Bouchelouche P. Bouchelouche K. Cervigni M. Daha L. K. (2008). Diagnostic criteria, classification, and nomenclature for painful bladder syndrome/interstitial cystitis: an ESSIC proposal. Eur. Urol. 53 (1), 60–67. 10.1016/j.eururo.2007.09.019 17900797

[B27] Vieira V. M. Hoffman K. Shin H. M. Weinberg J. M. Webster T. F. Fletcher T. (2013). Perfluorooctanoic acid exposure and cancer outcomes in a contaminated community: a geographic analysis. Environ. Health Perspect. 121 (3), 318–323. 10.1289/ehp.1205829 23308854 PMC3621179

[B28] Wang P. Liu D. Yan S. Liang Y. Cui J. Guo L. (2022). The role of ferroptosis in the damage of human proximal tubule epithelial cells caused by Perfluorooctane sulfonate. Toxics 10 (8), 436. 10.3390/toxics10080436 36006114 PMC9414058

[B29] Wang Z. Zang L. Ren W. Guo H. Sheng N. Zhou X. (2023). Bile acid metabolism disorder mediates hepatotoxicity of Nafion by-product 2 and perfluorooctane sulfonate in male PPARalpha-KO mice. Sci. Total Environ. 876, 162579. 10.1016/j.scitotenv.2023.162579 36870486

[B30] Wang Y. Hu J. Wu S. Fleishman J. S. Li Y. Xu Y. (2023). Targeting epigenetic and posttranslational modifications regulating ferroptosis for the treatment of diseases. Signal Transduct. Target Ther. 8 (1), 449. 10.1038/s41392-023-01720-0 38072908 PMC10711040

[B31] Wu S. Yuan T. Fu W. Dong H. Zhang Y. Zhang M. (2023). Perfluorinated compound correlation between human serum and drinking water: is drinking water a significant contributor? Sci. Total Environ. 873, 162471. 10.1016/j.scitotenv.2023.162471 36842602

[B32] Yan S. Ma H. Ren Y. Wang P. Liu D. Ding N. (2025). Perfluorooctane sulfonate causes HK-2 cell injury through ferroptosis and endoplasmic reticulum stress pathways. Toxicol. Ind. Health 41 (2), 73–82. 10.1177/07482337241300722 39560653

[B33] Yan H. Yin C. Xu F. Li R. (2026). Unraveling the connection between PFOA and bladder cancer: a study integrating network toxicology, molecular docking, and experimental validation. Toxicol. Appl. Pharmacol. 511, 117784. 10.1016/j.taap.2026.117784 41794187

[B34] Yang Y. Xie L. Zhu Y. Sheng Y. Wang J. Zhou X. (2023). Perfluorooctane sulfonate (PFOS), a novel environmental pollutant, induces liver injury in mice by activating hepatocyte ferroptosis. Ecotoxicol. Environ. Saf. 267, 115625. 10.1016/j.ecoenv.2023.115625 39492174

[B35] Yang J. Wen X. Fu Y. T. Li C. Chang H. L. Lu Y. Y. (2025). NCOA4-dependent ferritinophagy drives perfluorooctanoic acid-triggered renal ferroptosis in chicken embryos. J. Hazard Mater 499, 140112. 10.1016/j.jhazmat.2025.140112 41109030

[B36] Yuan H. Li X. Zhang X. Kang R. Tang D. (2016). Identification of ACSL4 as a biomarker and contributor of ferroptosis. Biochem. Biophys. Res. Commun. 478 (3), 1338–1343. 10.1016/j.bbrc.2016.08.124 27565726

[B37] Zhang L. Zheng X. Liu X. Li J. Li Y. Wang Z. (2023). Toxic effects of three perfluorinated or polyfluorinated compounds (PFCs) on two strains of freshwater algae: implications for ecological risk assessments. J. Environ. Sci. (China) 131, 48–58. 10.1016/j.jes.2022.10.042 37225380

